# Proceedings from the 4^th^ NextGen Therapies for SJIA and MAS virtual symposium held February 13–14, 2022

**DOI:** 10.1186/s12969-023-00863-2

**Published:** 2024-01-05

**Authors:** Rashmi Sinha, Fabrizio De Benedetti, Alexei A. Grom, on behalf of the participants listed below, Rolla F. Abu-Arja, Edward Behrens, Hermine Brunner, Scott W. Canna, Elvira Cannizzaro, Shanmuganathan Chandrakasan, Randy Cron, Kyla Driest, Yukiko Kimura, Christopher Leptak, Daniel J. Lovell, Rebecca Marsh, Bénédicte Neven, Peter A. Nigrovic, Nikolay Nikolov, Karen Onel, Sampath Prahalad, Susan Prockop, Pierre Quartier, Johannes Roth, Grant Schulert, Juliana M. F. Silva, Sebastiaan J. Vastert, Donna Wall, Ulrike Zeilhofer, Pauline Acevedo, Ronny Bachrach, Laura Bogg, Leah Bush, Anna Carlson, Cappy Culicchia, Kari Cupp, Vincent Delgaizo, Zulayka Martinez, Regina Minerva, Luciana Peixoto, Robyn Rivera, Sarah Tronsdal

**Affiliations:** 1 Systemic JIA Foundation, Cincinnati, OH USA; 2 Division of Rheumatology, Ospedale Pediatrico Bambino Gesù, Rome, Italy; 3 Division of Rheumatology, Cincinnati Children’s Hospital Medical Center, University of Cincinnati, Cincinnati, OH USA

## Introduction

Refractory Systemic Juvenile Idiopathic Arthritis (SJIA) remains a major source of morbidity and mortality in children with pediatric rheumatic disease [1, 2]. Although several potential therapeutic targets have been recently identified, only few patients have been enrolled in ongoing clinical trials. The 4^th^ **“**NextGen Therapies for SJIA and MAS” virtual symposium organized by the “SJIA Foundation” and held February 13–14, 2022**,** brought together SJIA families, leading researchers, clinicians and representatives from Pharma and FDA with the main goals to identify barriers to clinical research in this area and develop new strategies to advance it.

Systemic Idiopathic Arthritis (SJIA; estimated U.S. prevalence of 1:10,000 children) is the most severe subtype of chronic childhood arthropathy, notable for marked systemic immune activation with features of autoinflammation, and development of severe joint damage [2–6]. The licensing of IL-1 and IL-6-blocking monoclonal antibodies in 2012 resulted in superior control of SJIA-associated arthritis and systemic manifestations [7–11]. However, despite the use of these biologic disease modifying antirheumatic medications (b-DMARDs) about 15% of patients with SJIA develop macrophage activation syndrome (MAS), i.e. potentially fatal episodes of systemic hyperinflammation that are associated with hemophagocytosis [5, 6, 12, 13]. Furthermore, since the licensing of these b-DMARDs, children with SJIA (~ 5% in 2020) have increasingly been diagnosed with life-threatening chronic parenchymal lung disease (SJIA-LD) [14–16]. This has prompted speculations that drugs blocking IL-1 and/or IL-6 might contribute to the development of SJIA-LD [16]. Given high mortality and frequent need for hospitalization, there is an urgent need for life-saving treatments for SJIA-LD and MAS.

Translational research identified IFN-γ as the pivotal cytokine in MAS [6, 17–19] and Phase II clinical trials are in progress [20]. In contrast, the nature of the SJIA-associated lung disease remains elusive, and potential therapeutic targets in this disease still need to be defined. The typical histopathology of SJIA-LD includes (a) extensive lymphoplasmacytic inflammatory infiltrates in the lung interstitial tissue; (b) organizing pneumonia; (c) pleural and interlobular septal collagenous fibrosis; (d) vasculopathy associated with muscular mural thickening of the pulmonary arteries; and (e) alveolar inflammation with accumulation of foamy *alveolar macrophages* with some features of pulmonary alveolar proteinosis (PAP) [15, 16]. Features of PAP typically result from dysfunction of *alveolar macrophages* leading to accumulation of pulmonary surfactant in alveolar space, and it has been suggested that there may be acquired *alveolar macrophages* dysfunction in SJIA-LD.

Recent studies suggested potential risk factors for SJIA-LD: (a) SJIA onset under age 2 years with predominantly systemic features and limited arthritis, (b) recurrent MAS episodes, (c) anaphylaxis-like reactions to b-DMARDs, (d) highly pruritic rash associated with peripheral eosinophilia, (e) highly elevated serum IL-18 levels, and (f) and HLA DRB1*15 alleles [15, 16]. Gene expression profiling in SJIA-LD lung tissue revealed strong IFN-induced signature and numerous markers of T cell activation (predominantly Th1) [15]. Further, murine experiments showed that T-cell restricted overexpression of T-bet, a master transcription factor that drives production of the hallmark Th1 cytokine IFN- γ induces dysfunction of bone marrow macrophages, resulting in erythrophagocytosis locally, and *alveolar macrophages* dysfunction with the development of PAP-like lung pathology [21]. Together this suggests that either MAS alone or when combined with IL-1 or IL-6 blocking b-DMARDs contribute to the development of SJIA-LD. Alternative possibilities include environmental factors like infections that could be favored by inhibition of IL-1 and IL-6 with biologics. Marked differences in the prevalence of SJIA-LD in different geographic areas with similar patterns of utilization of b-DMARDS as well as large numbers of SJIA-LD cases reported in areas where b-DMARDs are not commonly used (China, India) support this hypothesis. Alternatively, high rate of anaphylaxis-like reactions as well as pruritic rash and peripheral eosinophilia observed in SJIA-LD patients point to the possibility of a delayed type hypersensitivity (DTH) reaction. The recently reported strong association between LD in SJIA and HLA DRB1*15 alleles, supports this hypothesis [22]. DTH may be driven by IFN- γ in response to either the monoclonal antibody (b-DMARD) itself or to the common excipient shared by these drugs (e.g., polysorbate). These possibilities create much apprehension among pediatric rheumatologists and families of children with SJIA worldwide whether anti-IL-1 and IL-6 b-DMARDs are truly safe for use in SJIA.

Further advancement of clinical studies focused on this patient population, however, has been hampered by the lack of agreement on whether SJIA patients with the lung disease should be classified as a separate diagnostic entity. In part, this is because arthritis is often absent in these patients while the systemic component of the disease seems more prominent compared to what has been historically seen in SJIA. As of now, the absence of arthritis in many of these patients precludes the definitive diagnosis of SJIA rendering them ineligible for existing clinical trials in this disease. Furthermore, the existing outcome measures typically used in clinical trials in SJIA are focused mainly on arthritis and do not fully capture the systemic component, specifically the lung disease.

Discussions around this topic at the 4^th^ “NextGen Therapies for SJIA and MAS” virtual symposium are captured by Nigrovic, et al. in Part 1 of the Proceedings. Furthermore, the existing outcome measures typically used in clinical trials in JIA are focused mainly on arthritis and do not fully capture the systemic component, particularly the lung disease.

In Part 2, Drs. Grom and Schulert summarized further discussions that defined emerging distinct clinical patterns of refractory SJIA. Considering the life-threatening nature of MAS and SJIA-LD, traditional placebo controlled/withdrawal design clinical trials may not be ethically acceptable in these patient populations.

In Part 3, Dr. Fabrizio de Benedetti summarized discussions focused on alternative designs that could be acceptable to clinicians, parents, and regulatory authorities.

Since strikingly high of serum IL-18 levels appear to be a unifying feature of various subgroups of refractory SJIA, Dr. Canna and colleagues explored the possibility of incorporation this biomarker as an inclusion criterion and measure of treatment response to a drug candidate (Part 4).

In the meantime, the patients with refractory SJIA continue to experience not only high mortality but also profound disease- and medication-related morbidities that lead to permanent tissue damage, with long lasting detrimental effects on all aspects of the child’s quality of life. The degree of immunosuppression needed in most of these patients is unlikely to be sustainable in the long term due to increased risk of infection and malignancy. As the optimal drug management of SJIA-LD remains elusive, hematopoietic stem cell transplantation (HSCT) has emerged as a potential alternative strategy in the interim. In Part 5, Dr. Silva and colleagues reviewed the growing experience with HSCT specifically for patients with SJIA and summarized the discussion about the technical aspects, optimal timing and preliminary outcomes for several patients who underwent HSCT. This description includes patients who have not been previously reported.

## Data Availability

All the data discussed during the meeting have now been published and appropriately referenced at the end of the manuscript.

## Abbreviations

b-DMARDs: Biologic disease-modifying anti-rheumatic drug
DTH: Delayed type hypersensitivity
HSCT: Hematopoietic stem cell transplantation
IFN-γ: Interferon Gamma
IL: Interleukin
LD: Lung disease
MAS: Macrophage activation syndrome
PAP: Pulmonary alveolar proteinosis
SJIA: Systemic Juvenile Idiopathic Arthritis

## References

[1] Canna SW, Schulert GS, de Jesus A, Pickering A, Brunner H, Gadina M, Levine S, Goldbach-Mansky R, Boutelle J, Sinha R, DeBenedetti F, Grom AA; NextGen 2019 Participants. Proceedings from the 2^nd^ Next Gen Therapies for Systemic Juvenile Idiopathic Arthritis and Macrophage Activation Syndrome symposium held on October 3–4, 2019. Pediatr Rheumatol Online J 20205;18(1):53. PMID: 32664935; PMCID: PMC7360380. 10.1186/s12969-020-00444-7 PMC7360380 32664935

[2] Petty RE (2004). International league of associations for rheumatology classification of juvenile idiopathic arthritis: second revision, Edmonton, 2001. J Rheumatol.

[3] Oen KG, Cheang M. Epidemiology of chronic arthritis in childhood. Semin Arthritis Rheum. 1996;26:575–91. 10.1016/s0049-0172(96)80009-6 8989803

[4] Mellins ED, Macubas C, Grom AA (2011). Pathogenesis of systemic juvenile idiopathic arthritis: some answers, more questions. Nat Rev Rheumatol.

[5] Ravelli A, Grom AA, Behrens EM, Cron RQ (2012). Macrophage activation syndrome as part of systemic juvenile idiopathic arthritis: diagnosis, genetics, pathophysiology and treatment. Genes Immun.

[6] Grom AA, Horne AC, De Benedetti F (2016). Macrophage activation syndrome in the era of biologic therapy: clues to pathogenesis and impact on diagnostic approaches. Nature Rev Rheumatol.

[7] Pascual V, Allantaz F, Arce E (2005). Role of interleukin-1 (IL-1) in the pathogenesis of systemic onset juvenile idiopathic arthritis and clinical response to IL-1 blockade. J Exp Med.

[8] Ruperto N, Brunner HI, Quartier P, Constantin T, Wulfraat N, Horneff G, Brik R, McCann L, Nistala K, Wouters C, Cimaz R, Ferrandiz MA, Flato B, Grom AA, Magnusson B, Ozen S, Abrams K, Kim D, Martini A, Lovell DJ (2012). Two randomized trials of canakinumab in systemic juvenile idiopathic arthritis. New Engl J Med.

[9] De Benedetti F, Martini A (1998). Is systemic juvenile rheumatoid arthritis an IL-6 mediated disease. J Rheumatol.

[10] Yokota S, Imagawa T, Mori M (2008). Efficacy and safety of tocilizumab in patients with systemic-onset juvenile idiopathic arthritis: a randomized, double-blind, placebo-controlled, withdrawal phase III trial. Lancet.

[11] De Benedetti F, Brunner HI, Ruperto N, Kenwright A, Ravelli A, Schneider WP, Wouters C, Zemel L, Burgos-Vargos R, Dolezalova P, Grom AA, Wulffraat N, Zuber Z, Zulian F, Lovell D, Martini A (2012). Randomized trial of tocilizumab in systemic juvenile idiopathic arthritis. New Engl J Med.

[12] Erkens R, Esteban Y, Towe C, Schulert G, Vastert S (2021). Pathogenesis and treatment of refractory disease courses in systemic juvenile idiopathic arthritis: refractory arthritis, recurrent macrophage activation syndrome and chronic lung disease. Rheum Dis Clin North Am.

[13] Grom AA, Ilowite NT, Pascual V, Brunner HI, Martini A, Lovell D, Ruperto N. Paediatric rheumatology international trials organisation and the pediatric rheumatology collaborative study group; Leon K, Lheritier K, Abrams K. Rate and Clinical Presentation of Macrophage Activation Syndrome in Patients With Systemic Juvenile Idiopathic Arthritis Treated with Canakinumab. Arthritis Rheumatol. 2016;68:218–28. PMID: 26314396. 10.1002/art.39407 26314396

[14] Kimura Y, Weiss JE, Haroldson KL, Lee T, Punaro M, Oliveira S (2013). Pulmonary hypertension and other potentially fatal pulmonary complications in systemic juvenile idiopathic arthritis. Arthritis Care Res (Hoboken).

[15] Schulert GS, Yasin S, Carey B, Chalk C, Do T, Schapiro AH, Husami A, Watts A, Brunner HI, Huggins J, Mellins ED, Morgan EM, Ting T, Trapnell BC, Wikenheiser-Brokamp KA, Towe C, Grom AA (2019). Systemic juvenile idiopathic arthritis-lung disease: characterization and risk factors. Arthritis Rheumatol.

[16] Saper VE, Chen G, Deutsch GH, Guillerman RP, Birgmeier J, Jagadeesh K, Canna S, Schulert G, Deterding R, Xu J, Leung AN, Bouzoubaa L, Abulaban K, Baszis K, Behrens EM, Birmingham J, Casey A, Cidon M, Cron RQ, De A, De Benedetti F, Ferguson I, Fishman MP, Goodman SI, Graham TB, Grom AA, Haines K, Hazen M, Henderson LA, Ho A, Ibarra M, Inman CJ, Jerath R, Khawaja K, Kingsbury DJ, Klein-Gitelman M, Lai K, Lapidus S, Lin C, Lin J, Liptzin DR, Milojevic D, Mombourquette J, Onel K, Ozen S, Perez M, Phillippi K, Prahalad S, Radhakrishna S, Reinhardt A, Riskalla M, Rosenwasser N, Roth J, Schneider R, Schonenberg-Meinema D, Shenoi S, Smith JA, Sönmez HE, Stoll ML, Towe C, Vargas SO, Vehe RK, Young LR, Yang J, Desai T, Balise R, Lu Y, Tian L, Bejerano G, Davis MM, Khatri P, Mellins ED, Childhood Arthritis and Rheumatology Research Alliance Registry Investigators (2019). Emergent high fatality lung disease in systemic juvenile arthritis. Ann Rheum Dis.

[17] Bracaglia C, de Graaf K, PiresMarafon D, Guilhot F, Ferlin W, Prencipe G, Caiello I, Davì S, Schulert G, Ravelli A, Grom AA, De Benedetti F (2017). Elevated circulating levels of interferon-γ and interferon-γ-induced chemokines characterize patients with macrophage activation syndrome complicating systemic juvenile idiopathic arthritis. Ann Rheum Dis.

[18] De Benedetti F, Prencipe G, Bracaglia C, Marasco E, Grom AA (2021). Targeting interferon-gamma in hyper-inflammation: opportunities and challenges. Nature Rev Rheumatology.

[19] Schulert GS, Pickering AV, Do T, Dhakal S, Fall N, Schnell D, Medvedovic M, Salomonis N, Thornton S, Grom AA. Monocyte and bone marrow macrophage transcriptional phenotypes in systemic juvenile idiopathic arthritis reveal TRIM8 as a mediator of IFN-γ hyper-responsiveness and risk for macrophage activation syndrome. Ann Rheum Dis. 2020;80:617–25. PMID: 33277241. 10.1136/annrheumdis-2020-217470 33277241

[20] De Benedetti F, Grom AA, Quartier P, Schneider R, Antón J, Bracaglia C (2019). Interferon-gamma (IFN-γ) Neutralization with emapalumab and time to response in patients with macrophage activation syndrome (mas) complicating systemic juvenile idiopathic arthritis who failed high-dose glucocorticoids abstract. Arthritis Rheumatol.

[21] Iriguchi S, Kikuchi N, Kaneko S, Noguchi E, Morishima Y, Matsuyama M, Yoh K, Takahashi S, Nakauchi H, Ishii Y. T-cell-restricted T-bet overexpression induces aberrant hematopoiesis of myeloid cells and impairs function of macrophages in the lung. Blood. 2015;125(2):370–82. 10.1182/blood-2014-05-575225 PMC4300389 25349175

[22] Saper VE, Ombrello MJ, Tremoulet AH, Montero-Martin G, Prahalad S, Canna S, et al. Severe delayed hypersensitivity reactions to IL-1 and IL-6 inhibitors link to common HLA-DRB1*15 alleles. Ann Rheum Dis. 2022;81:406–15. 10.1136/annrheumdis-2021-220578 PMC10564446 34789453

